# Prosthetic embodiment: systematic review on definitions, measures, and experimental paradigms

**DOI:** 10.1186/s12984-022-01006-6

**Published:** 2022-03-28

**Authors:** Jan Zbinden, Eva Lendaro, Max Ortiz-Catalan

**Affiliations:** 1 Center for Bionics and Pain Research, Mölndal, Sweden; 2 Department of Electrical Engineering, Chalmers University of Technology, Gothenburg, Sweden; 3 Operational Area 3, Sahlgrenska University Hospital, Gothenburg, Sweden; 4 Department of Orthopaedics, Institute of Clinical Sciences, Sahlgrenska Academy, University of Gothenburg, Gothenburg, Sweden

**Keywords:** Embodiment, Prosthetics, Ownership, Agency, Body representation, Phenomenology

## Abstract

The term embodiment has become omnipresent within prosthetics research and is often used as a metric of the progress made in prosthetic technologies, as well as a hallmark for user acceptance. However, despite the frequent use of the term, the concept of prosthetic embodiment is often left undefined or described incongruently, sometimes even within the same article. This terminological ambiguity complicates the comparison of studies using embodiment as a metric of success, which in turn hinders the advancement of prosthetics research. To resolve these terminological ambiguities, we systematically reviewed the used definitions of embodiment in the prosthetics literature. We performed a thematic analysis of the definitions and found that embodiment is often conceptualized in either of two frameworks based on body representations or experimental phenomenology. We concluded that treating prosthetic embodiment within an experimental phenomenological framework as the combination of ownership and agency allows for embodiment to be a quantifiable metric for use in translational research. To provide a common reference and guidance on how to best assess ownership and agency, we conducted a second systematic review, analyzing experiments and measures involving ownership and agency. Together, we highlight a pragmatic definition of prosthetic embodiment as the combination of ownership and agency, and in an accompanying article, we provide a perspective on a multi-dimensional framework for prosthetic embodiment. Here, we concluded by providing recommendations on metrics that allow for outcome comparisons between studies, thereby creating a common reference for further discussions within prosthetics research.

**Supplementary Information:**

The online version contains supplementary material available at 10.1186/s12984-022-01006-6.

## Introduction

Throughout history, state-of-the-art technology has been purposed to develop prostheses that restore functional independence in circumscribed tasks, for example by emulating the function of specific tools to facilitate the reintegration of people with amputations into society [1, 2]. Recent technological progress has produced prostheses with increasingly faithful volitional control [2, 3] and sensory feedback [4, 5]. The implementation of closed-loop control in a clinically viable form [6, 7] has made the objective of developing neuroprostheses capable of replacing lost extremities seem more attainable. Consequently, the concept of prosthetic embodiment has become a central theme in prosthetics research [8], not only when evaluating psychosocial outcomes of prosthesis use and user experience [9–11], but also as a quantitative metric in peripheral nerve stimulation studies [12–14].

In addition, by restoring the sensorimotor loop and allowing for the systematic modulation of efferent and (re)afferent signals, neuroprosthetics offers the opportunity to investigate the rules underlying bodily awareness. Not surprisingly, prosthetics has become the object of a broader research interest spanning different disciplines, from philosophy [15, 16] to social [17] and cognitive sciences [18–20]. Although engaging with similar research questions, such as what it means for an artificial limb to become an object of self-recognition, different scientific fields naturally formalize research questions using field-specific terminology and by building on previous concepts that do not clearly map across different research areas. For example, beyond its use in the prosthetics field, the concept of embodiment has also been employed in philosophy to discuss how one experiences one’s self [21, 22], and in neuroscience when investigating how the brain represents the body in health [23, 24] and disease [25].

Owing to this cross-influence, the concept of embodiment has been popularized within the prosthetics and neural engineering literature without precision as to its meaning. The lack of shared understanding of the word can cause confusion in the research community and complicate comparisons of studies that use embodiment as a metric of success, without agreeing on how to measure it.

In this article, we first systematically reviewed the literature on prosthetics to analyze the concept of embodiment. In particular, we conducted a thematic analysis of the definition of embodiment which provided the necessary context to understand and discuss embodiment within prosthetics. The thematic analysis revealed that a common approach was to define embodiment with respect to the subjective experience resulting from using a prosthetic limb. We favor considering embodiment following this approach because subjective experiences can be studied scientifically using psychophysical or experimental phenomenological methods. Psychophysics is commonly associated with stimuli and perception, but the concept of empirically measuring and correlating brain states and sensory experience can also be applied to volition and action [26]. Therefore, in a psychophysical, or experimental phenomenological framework, embodiment can be divided into the sense of ownership and agency, which makes it quantifiable, thus providing operable outcome measures within artificial limb development. In an accompanying perspective article to this review [8], we propose a multi-dimensional framework for prosthetic embodiment furthering this discussion. Here, we concluded by performing a second systematic review, analyzing the experimental paradigms and their employed measures to assess ownership and agency, which then led us to propose a series of recommendations of how to measure these phenomena in clinical practice and translational research.

## Methods

### Search strategy

In this review we conducted two systematic searches of the literature. The first search dealt with the main research question of how embodiment is defined across the prosthetic literature. The need for the second systematic search emerged from the thematic analysis of the definition of embodiment and answered the question of how agency and ownership are assessed within prosthetics.

Both systematic searches were performed using three electronic databases, namely Scopus, Pubmed and Web of Science. The first search used the following search keys respectively: TITLE-ABS-KEY(embodiment) AND TITLE-ABS-KEY(artificial lim* OR prosthet*), (embodiment[Title/Abstract]) AND ((artificial lim*[Text Word] OR prosthet*)[Text Word]) and *TS* = (embodiment AND (artificial lim* OR prosthet*)) AND AK = (embodiment OR prosthet*) AND AB = *(embodiment)*. The search was carried out on October 19, 2021, and no chronological constraints were set.

The second search was performed on October 26, 2021, using the search keys: TITLE-ABS-KEY ((ownership OR agency) AND (artificial AND lim* OR prosthet*)) AND (LIMIT-TO (SUBJAREA, "ENGI") OR LIMIT-TO (SUBJAREA, "MEDI")), (ownership[Title/Abstract] OR agency [Title/Abstract]) AND (artificial lim*[Text Word] OR prosthet*[Text Word]), and TS = ((ownership OR agency) AND (artificial lim* OR prostet*)) AND AK = ((ownership OR agency) AND (artificial lim* OR prosthet*)) AND AB = ((ownership OR agency) AND (artificial lim* OR prosthet*)).

### Selection criteria

Due to the heterogeneity of the studies employing the concept of prosthetic embodiment it was not possible to establish a clear list of eligibility criteria for inclusion in the first systematic review. The results of the search in the three databases were checked for doubles and merged. All the titles were then screened, and studies clearly not related to the field of prosthetics were removed from the results. All abstracts of the accepted titles were then read and again, the studies not relevant to the research questions were discarded. Studies with accepted abstracts were then read in full length and used for the subsequent thematic analysis.

For the second systematic review, the search results were first checked for duplicates and then merged. The titles had to be related to prosthetics, ownership, or agency and the abstract further needed to be in English and describe ownership or agency being measured to meet the inclusion criteria. Then the previously obtained list of accepted publications on embodiment was merged into the ownership and agency search. Duplicates, reviews, book chapters, studies not relevant for prosthetics, and studies without measures were removed. From the remaining studies, the employed experimental paradigm, the used measures, and information about the study population were extracted and aggregated in a table.

The review of experiments included all paradigms listed in said table. For the review of measures, only measures performed in experiments including participants with amputation were considered. Another inclusion criterium was suitability for clinical practice, i.e., the measure needed to provide an indication of the magnitude of ownership or agency in an individual participant. This for example makes neural correlates derived from fMRI studies using mass univariate analysis generally unsuitable as the results are valid at a group-level and do not provide an indication of the magnitude of agency or ownership in the single subject. Lastly, only measures differentiating between ownership and agency were included.

### Thematic analysis

Thematic analysis was used to identify recurrent themes in the way embodiment is defined in the literature on prosthetics. This analysis aimed at identifying commonalities running through all the selected articles. We followed an inductive approach: repeated rounds of reading and paraphrasing/coding of the excerpts from all selected articles allowed us to derive the themes from the text itself rather than from prior theory or research. In practice, we followed the step-by-step procedure prescribed by Braun and Clarke [27]. We first familiarized ourselves with the content of the articles and identified the parts of the text expressing their adopted interpretation or definition of embodiment and possibly how they were assessing it. We then extracted the text relating to the definition of embodiment and reported the exact words of the authors in a grid, where every row represents a different paper. The quoted text was reported all in the same column and if more than one conceptualization of embodiment was found in a single paper, it was added in a separate row dedicated to the same paper. Once in the grid, all the excerpts of the text were coded in adjacent columns. Briefly, assigning a code consists essentially in examining the quoted text and labelling it with a word or short phrase in order to capture its essence and summarize its content [28]. Not only does coding reduce the text to its main features but it also highlights differences and similarities across different conceptualizations of embodiment, thus enabling the subsequent step: the search of themes. The process of identifying themes can be thought of as an investigation of patterns of commonality where the first-order codes are better characterized with second-order codes that can then be grouped under potential thematic headings. Once grouped under the themes, first-order codes were re-examined for consistency. This process provided both a clear illustration of each theme and some indication of its prevalence in the examined literature. The result of the thematic analysis is the definition of what each theme is about and what aspects of the definition it captures, in the form of an analytic narrative that goes beyond the mere description of text. The thematic analysis is presented in the results.

## Results

### Thematic analysis of the definition of embodiment in prosthetics

The systematic search carried out across the three chosen databases resulted in 245 unique articles (PubMed (n = 95), Scopus (n = 199) and Web of Science (n = 57)). Screening of titles and abstracts reduced the list to 107 papers included for further thematic analysis. Full-text reading led to 3 additional articles being removed because not accessible, while 15 articles that came across through further reading were deemed relevant to the research question and manually included. This resulted in a final list of 119 accepted articles. The full selection process is documented in Additional file 1.

From the reviewed literature, it emerges that within the prosthetics field several different definitions have been adopted evidencing a clear lack of consensus. Table 1 reports a non-exhaustive list of publications that make use of the concept of embodiment. The table includes the definition of embodiment identified in the article and shows the coding process conducted under the thematic analysis. The complete list of publications and coding is available in Additional file 2.

**Table 1:** Examples of definitions of embodiment used within the field of prosthetics and their subsequent coding and categorization into themes

Definition	Code	Theme
Perceptual embodiment of the prosthetic limb (the perceptual awareness of the prosthesis in relation to the body) into the body schema. [29]	Experience of inclusion in the body schema	Mixed (Phenomenology and Body representations)
Embodiment is the process by which patients with limb loss come to accept their peripheral device as a natural extension of self. [30]	Experience of the artificial limb as part of the self	Phenomenology
At the implicit level of body representations, an object is said to be embodied if some of its properties—or all of them—are processed in the same way as the properties of biological body parts. [31]	Exploitation of neural resources normally devoted to representation of body parts	Body representation
Embodiment can also be associated with a large range of subjective explicit feelings, including feelings of bodily ownership, feelings of bodily control, of bodily integrity, affective feelings, and so forth. [31]	Subcomponents of the experience of embodiment, also experiences	Phenomenology
The incorporation of a prosthesis into one’s body schema. [32]	Inclusion in the body schema	Body representation
Embodiment is the percept that something not originally belonging to the self becomes part of the body. [33]	Experiencing an external object as part of the body	Phenomenology

The definitions are presented in chronological order of publication

Several publications make use of the concept of embodiment without defining it (34 out of 120 articles analyzed, e.g. [34–38], etc.). Further, different views on embodiment are sometimes presented within the same article (e.g. [39–41], etc.) so that in the remaining 86 articles, 125 definitions of embodiment were identified and extracted. Of these definitions, some were implied by the context and not expressively given (e.g. *“we assessed the integration of the device into the body schema […] through measurements of prosthesis embodiment”* [42], or *“prostheses must promote true embodiment so that they can actually be perceived as part of the body rather than a “simple” tool”*[43]*,* etc.). Yet, regardless of whether implicit or explicit, taken together, definitions can be reconducted to two main recurring themes: embodiment is either 1) described in terms of the process that leads to integration of a foreign object into the pre-existent neural infrastructure supporting the body (e.g. *“body representations”, “body schema”,* etc.) and exploitation of those resources or 2) described in terms of its subjective experiential correlate (i.e. experimental phenomenology). A detailed analysis of the two themes is presented in the following section.

### Definitions based on body representations

Throughout the prosthetic literature, embodiment is frequently described as a foreign object becoming part of the native infrastructure that supports perception, action, and ultimately self-awareness. The concept has been often worded in terms of integration or incorporation in the body representation, which at times is unspecified (“*a robotic hand is incorporated into one’s body representation”*, [44]) and other times is specified as “body schema” *(“the incorporation of a prosthesis into one’s body schema”*, [45]) or “body image” (*“prosthetics like rubber hands are incorporated into the body image itself*”, [46]). A variation of this way of expressing such integration is to regard embodiment as exploitation of the same (neural) resources that normally support the biological limb (*“E [an object] is fully embodied if and only if all its properties are processed in the same way as the properties of one’s body*”, [22]). Roughly 40% (49 out of 125) definitions analyzed have been found to belong to this theme.

The embodiment framework based on body representations has its origin in a long history of neurological and neuropsychological investigations and posits the existence of one (or many) representation(s) of the body. Although it is popular to contextualize the construct of prosthetic embodiment within the body representation framework, there is currently no consensus on how to categorize body representations.

For example, even though the dyadic taxonomy of body schema and body image is prominent in the literature [21], the relationship between these two types of representations has a history of ambiguity [47] and is still debated [48]. There is, however, converging consensus on certain aspects of the body schema and body image categorization. Specifically, the body schema is understood as the action-guiding sensorimotor representation [49]; it is highly plastic and can be updated during action [50] as it contains information on body parts needed for online action control [51]. Conversely, non-action-related perceptual and conceptual representations are included in the body image [49], which encompasses the emotion-, thought-, and belief-related representations of our body [21]. Generally, a persistent representation of the body structure is stored within the body image [51]. Alternatives to the dyadic body image/body schema categorization have also been proposed: refer to Schwoebel and Coslett [52] for an example of a triadic body representation model and to Longo [53] for an overview of six types of body representation.

Not only is there still a debate on the nature and function of body representations, but also it has been shown that handheld tools can also modify and become part of the body representations [54], thus making it harder to define the special way in which prostheses are embodied.

### Definitions based on experimental phenomenology

Another common way in which prosthetic embodiment, found in roughly 56% of the definitions (71 out of 125) considered in this analysis, has been defined is through the description of the subjective experience of an artificial limb perceived as if it was the biological one, which has also been widely used as an outcome in several qualitative studies [9–11, 55]. However, embodiment is a complex and multifaceted experience that is hard to fully capture due to the challenges of using language to describe, interpret, or assess, sensory experiences [56]. An endeavor that by itself feeds into a larger body of epistemological inquiry on how to properly account for the linguistic representation of our sensory perception and of the way we interact with the environment [57].

Attempts to operationalize the phenomenological notion of embodiment have led to its decomposition into subcomponents of this experience, which are easier to univocally describe and measure [58, 59]. Such subcomponents, representing cognitive proxies, include the sense of agency (hereinafter referred to as *agency*) and the sense of ownership (hereinafter referred to as *ownership*).

Other authors have suggested additional subcomponents of embodiment, for example, Longo et al*.* [58] studied the location of the embodied object respective to the location of the experience sensory feedback, and argued that embodiment could arise from spatial-representational mechanisms, or (sense of) *location*. As we discuss in an accompanying article to this review [8], ownership and agency can be mediated by certain basic principles, one of which considers spatial constraints. For example, misalignment between the biological hand and the object to be embodied can reduce perceived ownership [60–62]. Similarly, angular deviations between observed movement and the actual movement can decrease agency [63]. Thus, in this article, we consider all spatial-representational mechanisms as mediators of ownership and agency, instead of a separate subcomponent of embodiment. This decision is further supported by Bekrater‑Bodmann’s psychometric characterization of embodiment [59], where only ownership and agency fulfilled the significance threshold of the principal axis factoring. A spatial-representational subcomponent was relevant only when specifically targeting a three-factor solution.

Ownership is the sense that parts of our own body belong to ourselves. If our body is moving, ownership is also the perception that it is our body that is moving. On the other hand, agency is the understanding that we are the initiator of the action and in control of the movement (volition). Ownership and agency are aspects of self-awareness [64].

Understandings of embodiment as integration in body representation or as bodily aspects of human subjectivity are two sides of the same coin and some of the definitions that we have analyzed (roughly 4%, or 5 out of 125) reflect this falling under a mix of the two themes (e.g., “the individuals' feeling that a virtual or robotic limb is integrated in their own body scheme” [65]).

**Fig. 1:**
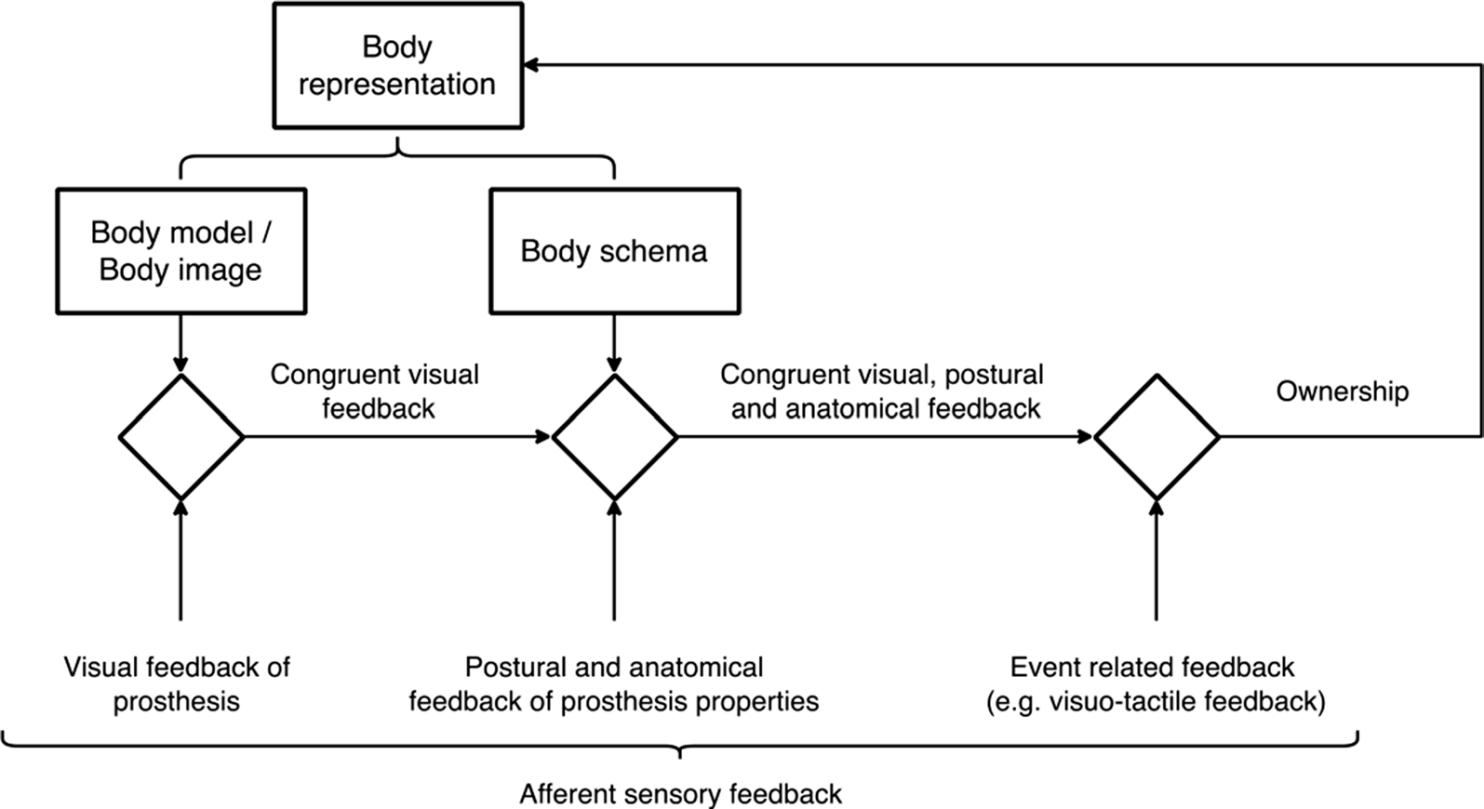
Neurocognitive model of the emergence of ownership: First the visual resemblance of the prosthesis is compared to the persistent representation of how a biological limb should look like in the body model. Successively, the postural and anatomical feedback of the prosthesis properties are compared to the current estimated postural state of the body, stored in the body schema. The last step is the sensory integration of the remaining afferent feedback. If there is consistency in all three comparator stages, ownership arises, and it is later used to update the body representation

Theories of the emergence of ownership and agency also capture this link. For example, in a model for ownership (see Fig. 1) put forward by Tsakiris [66], parts of the afferent sensory feedback related to the prosthesis are consecutively compared in a three-step comparator process.

**Fig. 2:**
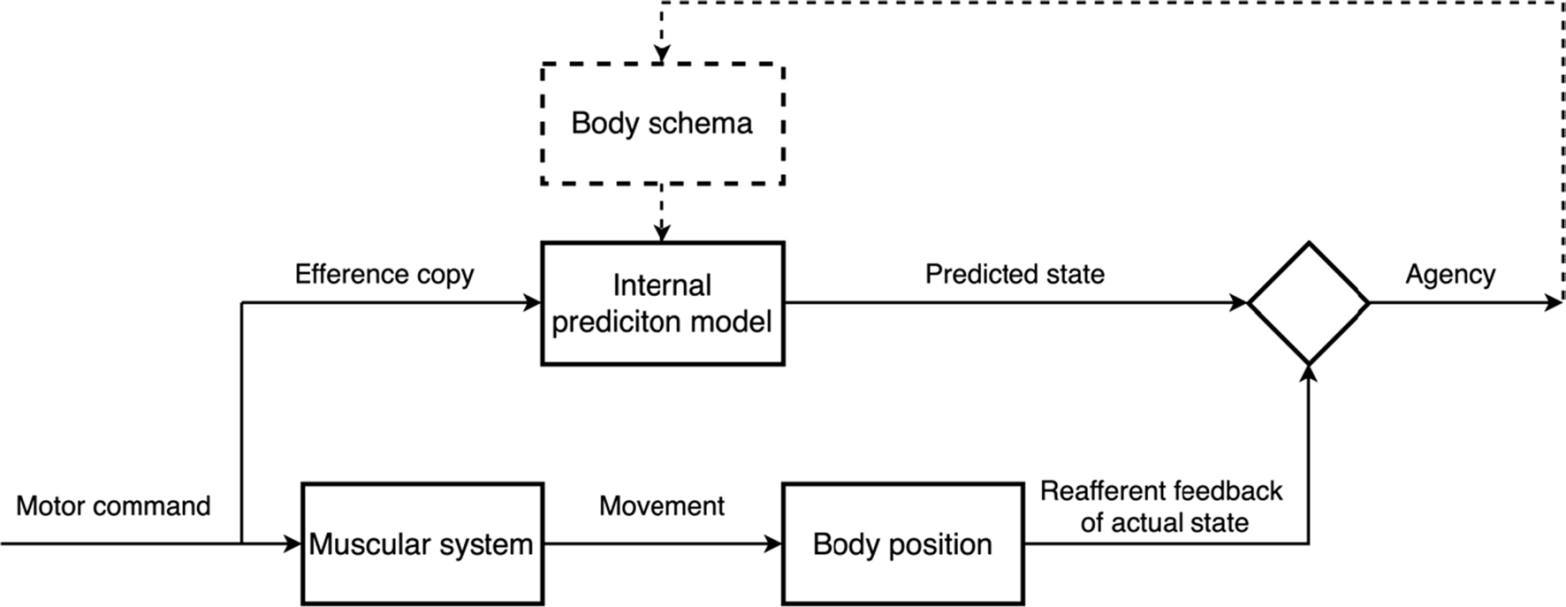
Comparator model of the emergence of agency: When a motor command is generated, an efference copy is sent to the internal prediction model. If the predicted state is congruent with the reafferent feedback of the actual body movement triggered by the motor command, agency arises. The dashed section is an addition to the commonly reported comparator model [70, 71] proposed by Martel et al. [72]. They reason that even though the comparator model was initially proposed as a theory of motor learning, it is highly complementary in respect to body representation as the internal model interacts with the body schema and the sense of agency

Similarly, the emergence of agency has also been described with a popular comparator model (see Fig. 2) proposing that a prediction model is implemented in the motor representation of one’s body [67, 68]. For a summary of alternative emergence theories of ownership and agency see Braun et al*.* [69].

### Experiments investigating ownership and agency

The systematic search carried out across the three chosen databases resulted in 772 articles (PubMed (n = 117), Scopus (n = 331) and Web of Science (n = 274)). Removal of duplicates (n = 126) and removal of papers with non-suitable titles (n = 512) and abstracts (n = 26) reduced the list to 84 papers. All publications from the previous embodiment search were added (n = 126), and the duplicates were removed (n = 20). Full-text reading aimed at identifying measures of ownership or agency led to a final list of 98 accepted articles. The full selection process is documented in Additional file 3.

The reviewed literature features three main experimental approaches used to investigate ownership and agency: experiments based on the rubber hand illusion (RHI) (n = 61), interviews (n = 9), and experiments with prosthetics in the loop (n = 28) (see Additional file 4).

### Rubber hand illusion paradigm

The most frequently used paradigm to study ownership and agency was the RHI, based on Botvinick and Cohens seminal work [73]. In their original RHI experiment, a rubber hand is placed in full view of a study participant. The participant’s corresponding hand is hidden from view. Two brushes are used to stroke and stimulate both the rubber- and the participant’s hand. Keeping the real hand immobile excludes agentic experiences and puts the focus on afferent signals. A well-established result of the RHI experiment is that synchronous visual and tactile stimuli applied in congruent locations elicit an illusory sensation of ownership towards the rubber hand. Asynchronous stimuli however do not lead to the emergence of ownership [74]. Worthy of notice is that about a third of the population is immune to the illusion or at most experience a weak illusion [60, 75–77].

Ehrsson et al*.* were the first to expand their ownership research to include participants with amputation [78, 79]. In their experiment, they provided tactile stimulation on the area of the residual limb that mapped to a digit on their phantom hand. In participants where such phantom maps could not be produced, the distal stump was stimulated. In a similar experiment, D’Alonzo et al*.* used a vibrotactile device or a brush to stimulate locations on the residual limb that refer to the digits of the hand, while brushing a rubber hand [80]. On average, stimulation with the brush led to equally high ownership ratings as in the original RHI condition, and to slightly lower ratings in the vibrotactile condition. Marasco et al*.* expanded on the paradigm by exchanging the brush stimulation with a pressure actuator in their RHI experiment with two target reinnervated participants with trans-humeral amputation [81]. Both reported ownership in the condition where the visual feedback was both spatial and temporally congruent with the percept caused by the pressure actuation on the reinnervated skin.

Recent studies investigated direct nerve stimulation as an alternative to elicit the RHI. On two participants with trans-radial amputation, Rognini et al*.* used intrafascicular electrodes to administer neurotactile simulation while providing visual feedback of the percept location via a head-mounted display [14]. Both participants reported ownership towards the virtual prosthesis. In an experiment in four participants with transhumeral amputation who were implanted with a neuromusculoskeletal prostheses [6], our group used synchronous tapping and peripheral nerve stimulation to administer the RHI with the prosthesis connected to the participant’s body [77]. Despite referring to their prosthesis as part of their body in daily life [10], none of the participants reported ownership over their prosthesis using congruent visuo-tactile stimulation. However, none of the participants reported ownership during the original RHI experiment in their contralateral hand either, and therefore a potential explanation might be that these participants are part of a non-negligible subgroup of people not responding to the RHI at all.

Previous studies with able-bodied participants [74] extended the passive RHI paradigm to incorporate an active motor control task to study agency alongside ownership. Page et al*.* were the first to use the active RHI paradigm with a participant with upper-limb amputation [12]. Utah Slanted Electrode Arrays were implanted into one participant. Using these electrodes, neural stimulation could elicit sensory percepts that matched the location of the sensors in the participant’s prosthetic hand. Like the original RHI setup, a barrier was placed between the residual limb and the prosthesis. In different conditions, the participant either got to control the prosthesis without sensory feedback, an investigator manually pressed on the sensors in the hand with the control turned off, or the participant controlled the prosthesis in closed-loop with sensory feedback. In all three conditions increased ownership and agency compared to a purely visual condition were reported, respectively.

### Interviews and questionnaires

Questionnaires and interviews, mostly semi-structured ones, were another common paradigm. Participants were interviewed in written from [20, 59, 82], over phone [83], and face-to-face [10, 11, 83–86]. However, most of the interviews investigated a more general experience of living with an upper-limb amputation and an artificial limb (e.g., environmental aspects [11], sensory feedback [85], phantom-limb experience [82], and home-use [10, 86]. Nevertheless, participants reported ownership [20, 82], agency [85], or both [10, 86] over their prostheses during these interviews.

Two recent publications chose to have a stronger focus on ownership and agency in their interviews. Sturma et al. explicitly inquired about the participant’s ownership and agency towards their prosthesis after having undergone bionic reconstruction after a brachial plexus injury [84]. And Bekrater‑Bodmann let 118 participants with lower-limb amputation fill in a questionnaire with common questions aiming to inquire about ownership and agency to create the first validated questionnaire for assessing ownership and agency in prosthetic users [59].

### Prosthetics in the loop

This paradigm category aggregates multiple different, and mostly novel approaches to investigate ownership and agency—with the main common denominator that the prosthesis is in focus. Of all the experiments in this category including participants with amputation (n = 18), the most common paradigm (n = 6) to study ownership and/or agency was based on functional tests. Participants with upper-limb amputation carried out the box and blocks test and the Southampton Hand Assessment Procedure (SHAP) [13], performed a Virtual Egg Test (VET) [35], or discriminated between objects of different sizes and compliances [34]. For participants with lower-limb amputation, the functional tests included overground walking, stair tasks, and obstacle avoidance tasks [42, 87, 88]. In all instances, any ownership and/or agency measurement was performed post-hoc to the functional tests. In a similar post-hoc manner, ownership and agency were assessed after home-use of a prosthetic system [9, 89].

Recently, several studies proposed paradigms that explicitly focus on investigating ownership and agency for prosthetics, instead of them being a secondary ad-hoc research question. One such experiment, targeting ownership over a prosthesis, is the Prosthesis Incorporation (PIC) assessment [90]. The PIC is based on the cross-model congruency paradigm, which tests the ability of the study participants to ignore one form of feedback in favor of another form of feedback. In an experiment by Marasco and colleagues, two prosthetic users underwent targeted motor and sensory reinnervation [91]. The participants received tactile stimulation on the reinnervated skin either congruent or incongruent to visual feedback indicating the stimulation location while grasping an object. If the visual feedback location is congruent with the tactile feedback location, participants discerned the location of a stimulation faster compared to when they received incongruent feedback.

Marasco et al. also proposed an experiment with a focus on agency over a prosthetic limb, based on the Libet clock experiment [92]. Prior to the experiment, the nerve endings in the residual limb of participants with upper-limb amputation were surgically redirected to reinnervate the skin and muscles on the upper arm [93]. Vibrating the reinnervated residual muscle at 90 Hz in different locations, the authors induced kinesthetic percepts of digit flexion and extension. For the experiment, the participants were presented with an electromyography-controlled virtual prosthetic hand and told to touch a virtual ball. Touching the ball resulted in a stimulation corresponding to a cylinder-grip percept while playing a tone with random delay. The participants were asked to estimate the time delay. In conditions where intent and kinesthetic and visual feedback were congruent, the participants estimated shorter time delays compared to incongruent conditions.

Other noteworthy experiments to investigate ownership and agency study communicative gestures executed with the prosthesis [94], compare postural sway when the prosthesis is either donned or doffed [40], and investigate how one’s own prosthesis is represented in the brain by comparing pictures of one’s own prosthesis to the prosthesis of others [41, 95].

### Measures of ownership and agency in prosthetics

**Fig. 3:**
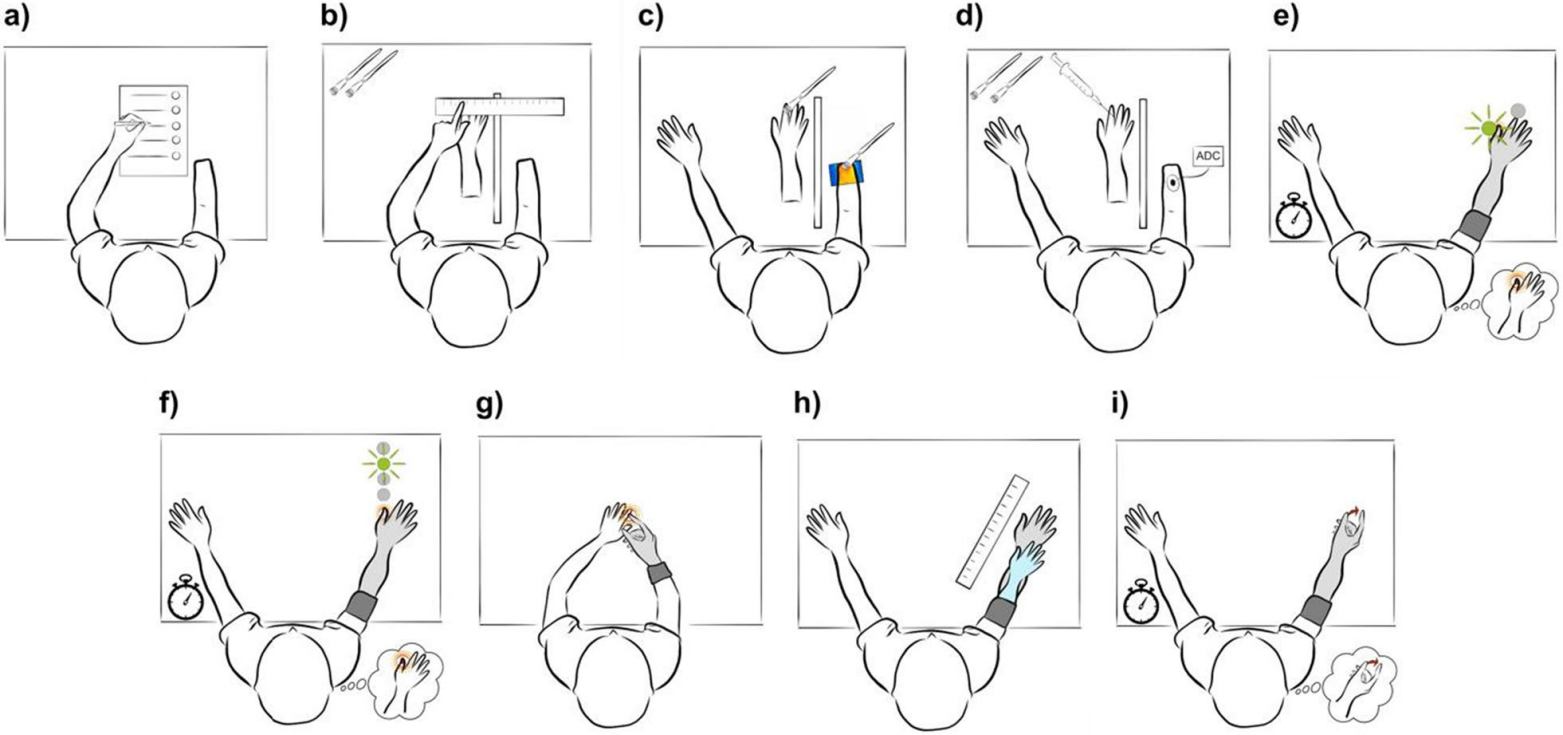
Illustration of the ownership and agency measures: **a** Explicit ownership and agency are measured by administrating a questionnaire. **b** The proprioceptive drift measures the distance between where e.g., a rubber hand that the participant experiences ownership for is perceived compared to where the phantom hand is perceived. **c** The skin temperature measure aims to capture a skin temperature change due to increased assessed using e.g., a thermal camera. **d** The galvanic skin response measures increase in sweating in case e.g., a rubber hand that the participant experiences ownership for is under threat. **e** In the crossmodal effect, the time difference between congruent and incongruent feedback is assessed to implicitly measure ownership. **f** A tactile distance perception task implicitly evaluates ownership by administering sensory stimulation while flashing a visual cue at different distances away from the percept location. **g** Applied force is measured to implicitly assess ownership in the sensory attenuation task, where force perception depends on whether the touch is self-administered or externally administered. **h** The normalization of the phantom limb to e.g., match the prosthesis was proposed to implicitly evaluate ownership over the prosthesis. **i** In the intentional binding task, implicit agency is assessed by estimating sensory feedback time delay after either voluntary or non-voluntary movements triggered the sensory feedback

This section summarizes the measures used in the above-described experiments (see Fig. 3 and Table 2). Both ownership and agency can be distinguished between the pre-reflective feeling of the experience and the a posteriori judgment of the experience (see for example Synofzik et al., who argue for a distinction between a feeling of ownership and agency and their judgments [71]). Subsequentially, agency and ownership can be measured explicitly and implicitly—a distinction we use to structure the following section.

**Table 2:** Overview of ownership and agency measures extracted from the literature research

Measure	Measured modality	Study population
Questionnaires (Ownership)	Explicit ownership	AB (n = 55), LAnP (n = 9), LAwP (n = 25)
Proprioceptive drift	Implicit ownership	AB (n = 30), LAnP (n = 4), LAwP (n = 1)
Temperature	Implicit ownership	AB (n = 4), LAnP (n = 1)
Galvanic skin response	Implicit ownership	AB (n = 12), LAnP (n = 2)
Cross modal congruency	Implicit ownership	AB(n = 2), LAwP (n = 1)
Tactile distance perception	Implicit ownership	LAwP (n = 2)
Sensory attenuation	Implicit ownership	AB (n = 2), LAwP (n = 1)
Phantom-limb length	Implicit ownership	LAwP (n = 3)
Questionnaires (Agency)	Explicit agency	AB (n = 17), LAnP (n = 1), LAwP (n = 11)
Intentional binding	Implicit agency	AB (n = 2), LAnP (n = 1), LAwP (n = 1)

Only measures performed in at least one study including participants with amputation and only measures explicitly related to either ownership or agency were included

AB, able-bodied; LAnP, participant with limb amputation not wearing a prosthesis, and LAwP, participant with limb amputation wearing a prosthesis)

### Explicit ownership and agency measures

The main measure to assess explicit ownership and explicit agency has been questionnaires. And often the same questionnaire inquiries about both explicit ownership and explicit agency—making a separation between explicit ownership and agency measures unpractical.

The only questionnaire that investigates only one of them, namely ownership, was the original rubber hand illusion (RHI) questionnaire, introduced together with the RHI experiment itself [73]. Conventionally, three questions inquiring into the causation and location of the stimulus as well as the association of the rubber hand to one’s body have been used to assess explicit ownership. Commonly, the answers are rated on a 7-point Likert scale. The RHI paradigm was later adapted to include a moving hand [74] and consequently, the questionnaire was expanded to include questions assessing explicit agency. Most commonly, such combined questionnaires are based on Longo et al*.*’s [58] work, where a psychometric analysis (*i.e.*, principal component analysis) was performed to identify the latent factor structure underlying the RHI experience in non-amputated participants. Recently, several new questionnaires have been proposed aimed to generally assess explicit ownership and explicit agency in prosthetics instead of only within the RHI paradigm. Graczyk et al*.* developed the Patient Experience Measure (PEM), covering a wide range of common experiences as a prosthetic user [9]. The focus of the PEM lies on explicit ownership and the consequent impact on body image, as well as questions on the agentic experience in terms of self-efficacy and efficiency during prosthetic use. A similar questionnaire was developed by Gouzien et al*.* [31]. They used four categories (quantity of use, functional use, aesthetic use, and psychological use) to infer a bodily integration score. Furthermore, Bekrater-Bodmann and colleagues recently developed and validated the Prosthesis Embodiment Scale for Lower Limb Amputees (PEmbS-LLA) [59], as well as the Prosthesis Embodiment Scale for Upper Limb Amputees (PEmbS-ULA) [96], both focus on explicit ownership, explicit agency, and anatomical plausibility.

The use of the questionnaire has also been subject to criticism: As the questionnaire forces the participants to provide a retrospective judgment of the experiment, it has been questioned whether the reports correspond to the actual vividness of the experience, or rather on how confidently they judged the experience to be [22]. It was also shown that preconditioning by phrasing influences the measurement outcome: RHI study participants rated e.g. ﻿the *feeling* that the rubber hand is part of their body higher compared to the *belief* that the rubber hand is part of their body [97].

Aiming for a measure with less bias than questionnaires, Chancel and Ehrsson [98] recently introduced a two-alternative forced-choice psychophysics task to assess explicit ownership. However, this approach has not yet been tested with participants with amputation.

### Implicit ownership measures

The most used measure to assess implicit ownership is the so-called proprioceptive drift. During the RHI with able-bodied participants, the sensed hand position can shift towards a rubber hand. This shift is measured via a pointing task where the participant indicates the perceived location of e.g., the index finger on a ruler before and after the experiment. In experiments with participants with upper-limb amputation, the location shift of their perceived phantom is evaluated instead [78–80]. Despite the difference in proprioceptive drift between the synchronous and asynchronous RHI condition generally being a reliable measure of the RHI, proprioceptive drift as a measure of ownership has been criticized. Rohde et al*.* [99] observed that prolonged visuo-tactile asynchrony affected the perception of the hand location in general and thus concluded that the absence of proprioceptive drift in the asynchronous RHI condition is caused by the asynchronous stimulation itself. Further, it was shown that external manipulation of a study participants hand position affected the reported proprioceptive drift, but not the reported ownership over a rubber hand [100].

Skin temperature of the stimulated arm, measured via thermistors or a laser thermometer, was reported to decrease during a RHI experiment with able-bodied participants [101]. Based on these findings, the authors suggested that the body downregulates metabolic efforts in the hand as the sense of ownership shifts away from one’s own biological limb, making skin temperature changes another measure for implicit ownership. Conversely, an increase in temperature in the usually colder residual limb compared to the contralateral limb was reported for one participant with trans-humeral amputation [81]. Here it was proposed that temperature normalization was due to the perceived ownership over the rubber hand. Consistent skin temperature changes during the RHI have, however, been difficult to reproduce and the correlation of temperature change to ownership has been questioned [102, 103].

An increase in psychologically-induced sweating was measured when a limb with strong ownership association within the peripersonal space was exposed to a threat in both able-bodied participants [104] and participants with upper-limb amputation [78, 80]. Thus, changes in skin conductance levels, determined with galvanic skin response sensors, were suggested as yet another implicit ownership measure.

The crossmodal congruency effect (CCE) is a further option for measuring implicit ownership. In a typical crossmodal congruency task for assessing upper-limb implicit ownership, touch feedback to two locations on the hand [105, 106] or on the skin of a reinnervated residual limb [91] are provided together with two visual distracters. The visual distractors light up either congruent or incongruent with respect to the perceived touch location. Participants generally indicate the location of the touch feedback faster when the stimulation and visual distractor location coincide. The indication time difference between congruent and incongruent feedback is the CCE. The CCE is known to depend on the distance between the tactile and visual stimuli and can thus be used to investigate multisensory interactions with respect to peripersonal space which is closely linked to ownership [107].

Like in a crossmodal congruency task, implicit ownership can be investigated via peripersonal space integration doing a tactile distance perception task. For this measure, participants react as fast as possible to a sensory stimulus while seeing a visual distractor at different distances of the perceived stimulus [33, 108]. A reaction time vs. distance profile like able-bodied performance while wearing the prosthesis compared to receiving the feedback on the stump suggested that the peripersonal space increased to include the prosthesis.

Sensory attenuation, or the decrease of perceived intensity of a sensation caused by self-generated movement [109], was reported to be determined by ownership [110]. Sensory attenuation can be assessed by a force-matching task, where participants receive either a self-administered or external, yet known, force stimulus and then they are subsequently prompted to generate the same perceived force by e.g., pressing their index finger against a force sensor. Fritsch et al. showed that sensory attenuation can also be elicited in upper-limb prosthetic users when they touch their foot with their prosthesis [96]. They further reported increased sensory attenuation with higher self-reported explicit ownership over the prosthesis, thereby positioning sensory attenuation as another implicit ownership measure.

One implicit ownership measure is exclusive for study participants experiencing a phantom limb due to amputation. Namely, measuring phantom-limb length, where the perceived length of the phantom is measured before and after the intervention. The effect has been investigated with the RHI [14, 35] and in follow-up tests after prosthetic home use [9] in participants with upper-limb amputation and has been shown to correlate with explicit ownership over the prosthesis. Estimating actual limb length instead of phantom-limb length did, however, not result in conclusive correlates for ownership [39].

### Implicit agency measures

The main measure used to evaluate implicit agency is intentional binding as initially explored within the Libet clock paradigm [111]. Participants of this experiment pressed a button which resulted in auditory feedback. If the movement was executed voluntarily, participants perceived the time interval between their action and the result of their action to be shorter than when the movement was executed involuntarily—the two cues are temporally bound together in consciousness. The original time estimation method, where participants read times of a clock, was later replaced by a direct interval estimation procedure in which participants simply report the perceived interval (e.g., in ms) [112]. This measure can readily be incorporated in experiments including prosthetic users, where voluntary prosthetic movements can be decoded from e.g., myoelectric signals, and involuntary movements can easily be executed by sending external motor commands to the prosthesis (instead of having to use transcranial magnetic stimulation [111]). The illusion of involuntary movement can also be elicited by a prosthetic system that can provide proprioceptive feedback [92].

## Discussion

### Towards a working definition of prosthetic embodiment

The plethora of terms and definitions surrounding embodiment stands as a major reason for misunderstanding and complications in the comparisons of studies that use embodiment as a metric of success within prosthetics. From the analysis of the definitions of embodiment used within the prosthetic literature, two recurrent themes emerged: embodiment can be understood as grounded in body representations or phenomenology.

Prosthetic embodiment defined within both the body representation and the phenomenological framework is dependent on, and thus interconnected via, the individual experiences of ownership and agency. When it comes to research on prosthetics, where embodiment is used as a metric of success, considering embodiment in the context of body representations is problematic. For example, without clear agreement on what type of body representation (e.g., body image, body schema, etc.) is relevant for the incorporation of an artificial limb, different routes for verifying successful embodiment might be pursued depending on the criteria needed to satisfy the specific definition adopted, thus arriving at unclear or contrasting results. The question of whether the prosthesis is embodied is then set back on a level that is challenging to prove or falsify. Similarly, views of embodiment as “the processing of the properties of an object in the same way as the properties of one’s body” [22] or by paraphrasing as “the successful allocation of brain resources, originally devoted to controlling one’s own body, to represent and operate external objects” [113] set the problem at a higher level of complexity, which requires the additional determination of how neural resources are normally deployed when experiencing awareness of one’s own limb.

For the practical purposes of assessing whether an artificial limb is embodied, measures more directly based on how the user relates to the device are more amenable. Ownership and agency, cognitive proxies of the experience of embodiment, lend themselves well to a working definition because they limit the scope of the investigation to specific aspects of the experience.

### Experiments and measures for assessing prosthetic development

#### Suggested measures for the RHI paradigm

The RHI experiment has a long-standing history as the gold-standard experiment for studying ownership. For participants with upper-limb amputation, it has been successfully demonstrated that different sensory feedback strategies (sensory substitution [80], targeted sensory reinnervation [81], and direct nerve stimulation [12, 14]) also lead to increased ownership over a rubber hand or prosthesis within the RHI paradigm. We, therefore, deem the RHI experiment a suitable approach to investigate ownership, specifically for benchmarking different sensory feedback strategies when a closed-loop prosthetic system is not available. Especially in a non-agentic RHI setup where confounding variables potentially affecting ownership (such as algorithms for decoding motor volition or the proficiency in using a prosthetic device) are excluded.

As measures of explicit ownership for RHI experiments evaluating sensory feedback strategies, we suggest using an adaptation of the standard RHI questionnaire on a 7-point Likert scale (e.g., [78]) to allow for comparison to previous works. Furthermore, certain control questions (for example, referring to an additional limb or perceived location of a sensation) are prone to misinterpretation, especially in the case of participants with a phantom limb, and should therefore be omitted. It has even been pointed out that the use of the RHI control questions assessing suggestibility is lacking empirical support [114]. Since the actual control condition in the RHI is asynchronous stimulation, the control question could therefore be completely omitted, making the questionnaire easier to administer and its result more relevant.

As support for the explicit ownership measure within the RHI paradigm, we suggest using the cross-modal congruency effect, as it reflects the spatial imprecision of percepts generally present with current neuro stimulation approaches in its scoring. We specifically suggest using the adjusted CCE score specifically proposed for assessing advanced bionic feedback systems as it standardizes scores across participants and features a benchmark for comparison to the obtained results [90].

Proprioceptive drift can be used as another measure for implicit ownership in prosthetics, but only if the full phantom-limb movement is characterized and studied in detail. Since phantom limbs can be frozen in place, be perceived in anatomically impossible angles, or not be present at all, great care needs to be taken in comparing proprioceptive drift results with results from able-bodied experiments.

As for other implicit ownership measures traditionally used within the RHI paradigm, we suggest caution. The galvanic skin response to a threat to the rubber hand, while correlating with explicit ownership with participants with upper-limb amputation [78, 80], has yet to demonstrate enough resolution to allow for comparison between different sensory feedback strategies. Further, as Niederhuber et al*.* pointed out, the threat could be perceived towards the phantom limb instead of the prosthesis in case of spatial overlap [115]. Decrease in skin temperature during the RHI suffers in turn from reproducibility issues and was shown to not always correlate with ownership [102, 103].

Generally, for any RHI experiment with participants with amputation, we advocate to always have a condition where the participant has the prosthesis donned as they would during daily use and, if possible, a comparison to the contralateral limb.

### Suggested measures for experiments with prosthetics in the loop

Using a donned prosthesis blurs the line between the RHI paradigm and recent experiments with prosthetics in the loop to assess ownership. Especially when the prosthesis allows for voluntary execution of movements. Thus, instead of adding a dynamic element to the RHI as was done with able-bodied participants, we recommend using the dynamic Prosthesis Incorporation (PIC) assessment based on the CCE measurements instead [91]. Only such a closed-loop approach can determine if the investigated sensory feedback approaches are viable for home-use, or if the prosthetic system is limited by e.g., computational constraints. That is, multi-sensory integration (constraints from temporal binding windows [116, 117]) is a time-sensitive process, and thus, all the signal- and control processing must occur below human perception thresholds.

To assess implicit agency, we back the use of the intentional binding measure. Intentional binding has a long-standing history of reliably determining if movements were executed voluntarily. It already has been shown to correlate with explicit agency when proprioceptive feedback, a crucial component of the reafferent feedback necessary for agency to emerge, was added to a closed-loop prosthetic task [92]. We anticipate a similar correlation when comparing different prosthetic control algorithms, making intentional binding a useful measure both for the development of agency-related sensory feedback strategies, as well as for prosthetic control strategies.

In either case, measuring explicit ownership or explicit agency, the use of a questionnaire will help to capture the individual experience of the participant. Furthermore, having both the employed explicit and implicit measures agree with each other, provides for stronger support of inferences and conclusions. With a prosthesis in the loop, we suggest questionnaires designed explicitly for prosthetic use [9, 31]. Particularly the PembS developed by Bekrater-Bodmann and colleagues, as the questionnaire has been validated and specifically distinguishes between explicit ownership and explicit agency [59].

Such a questionnaire can also be employed during home-use trials, where participants tested novel algorithms for sensory feedback or control in their home environment. Here we advocate taking the temporality of both ownership and agency into account and suggest administering the questionnaire multiple times to obtain a more robust result [10]. The subjective longitudinal results of each participant can then be compared to determine the efficacy of the tested algorithm.

### Need for further standardization of experimental results

Currently, multiple measure outcomes suffer from the lack of standardization, complicating comparison across participants and research groups. This is not surprising as the population of people with amputation and their used prosthetic system is highly heterogenic. We, therefore, propose a list of measures and experiments for specific applications to facilitate comparison between research groups (see Table 3). In general, as the prosthetic field aims to fully restore both sensation and control after limb loss, we suggest to used able-bodied performance as a comparison for measure scores whenever possible, as used by e.g. Marasco et al. [91]. Further, validation of measures, as done by e.g., Bekrater-Bodmann and colleagues [59] is another important step in measure standardization and comparability. We thus encourage other authors to incorporate and thereby further validate recently proposed implicit ownership measures like phantom-limb length changes [14, 35], sensory attenuation [96], and tactile distance perception [33]. These measures, being accessible and low cost in their administration, would be valuable additions to the ownership measurement toolkit.

**Table 3:** Suggested measures and experiments for common evaluations in prosthetics to improve comparability between research groups

Application	Experiment	Measures
Evaluate sensory feedback strategy when closed-loop prosthetic system is not available	RHI	Explicit ownership: Adapted RHI questionnaire [78] only containing ownership question on 7-point Likert scale Implicit ownership: adjusted CCE score[90]
Evaluate sensory feedback strategy when closed-loop prosthetic system is available	(dynamic) Prosthesis Incorporation (PIC) assessment	Explicit ownership: PembS [59, 96] omitting agency questions if not dynamic version of PIC Implicit ownership: adjusted CCE score [90]
Evaluate control strategies	Adapted Libet clock	Explicit agency: PembS [59, 96], omitting ownership questions if no sensory feedback is provided Implicit agency: Intentional binding using a direct time interval estimation [92]
Evaluate novel sensory or control algorithms during daily life	Interview	Explicit ownership and eplicit agency: Administer PembS [59, 96] at regular time intervals

## Conclusions

In this article, we analyzed the different definitions of embodiment used within the prosthetics field. As a result, we identified two common categories within the definitions of prosthetic embodiment: embodiment grounded in body representations or experimental phenomenology. The latter allowed for a subdivision of prosthetic embodiment into ownership and agency, which lent itself to a pragmatic definition that allows the otherwise elusive construct of embodiment to be measured, and therefore we consider it as the preferred definition in the field of prosthetics. This is further justified in an accompanying article to this review where we introduce a multi-dimensional framework for prosthetic embodiment.

Here, we also offered a summary of different ownership and agency experiments and provided an overview of ownership and agency measures conducted with participants with amputation and prosthetic users. We compiled and discussed recommendations for both the measures and experiments with the objective to facilitate new studies on ownership, agency, and embodiment within the field of prosthetics.

This article offers a framework highlighting a pragmatic definition of prosthetic embodiment and suggested practices for experiments within the prosthetics field, and thereby creates a common reference for further discussion on advancing prosthetics research.

## Supplementary Information

**Additional file 1.** Results of systematic embodiment search.

**Additional file 2.** Summary of embodiment thematic coding process.

**Additional file 3.** Results of systematic ownership and agency search.

**Additional file 4.** Summary of ownership and agency experiments and measures.

## Data Availability

All data is available either as part of the article or as Additional files.
